# Supplementary material for: Safety and Non-Inferiority Evaluation of Two Immunization Schedules with an Inactivated SARS-CoV-2 Vaccine in Adults: A Randomized Clinical Trial
Source: Vaccines (Basel). 2022 Jul 6;10(7):1082. doi: 10.3390/vaccines10071082 (PMC9323976; doi:10.3390/vaccines10071082)
Supplement: Supplementary file 1 [file vaccines-10-01082-s001.zip › vaccines-1768725-supplementary.pdf]

Supplementary Table S1. Frequency of adverse events within first 60 min post vaccine administration, by dose and schedule.

	First dose (n=2302)		Second dose (n=2212)	
	Schedule 0-14 (n=1090)	Schedule 0-28 (n=1212)	Schedule 0-14 (n=1038)	Schedule 0-28 (n=1174)
**Local adverse events, n (%)**				
Pain	22 (2.02)	17 (1.4)	22 (2.12)	11 (0.94)
Induration	0 (0.00)	1 (0.08)	0 (0.00)	2 (0.17)
Pruritus	3 (0.28)	0 (0.00)	5 (0.48)	1 (0.09)
Redness	3 (0.28)	1 (0.08)	2 (0.19)	1 (0.09)
Swelling	0 (0.00)	0 (0.00)	0 (0.00)	1 (0.09)
**Systemic adverse events, n (%)**				
Headache	6 (0.55)	6 (0.5)	4 (0.39)	3 (0.26)
Fatigue	1 (0.09)	1 (0.08)	2 (0.19)	2 (0.17)
Muscle pain	0 (0.00)	1 (0.08)	0 (0.00)	1 (0.09)
Diarrhoea	1 (0.09)	1 (0.08)	0 (0.00)	1 (0.09)
Nausea	0 (0.00)	1 (0.08)	1 (0.10)	2 (0.17)
Anorexia	0 (0.00)	0 (0.00)	0 (0.00)	1 (0.09)
Arthralgia	0 (0.00)	0 (0.00)	0 (0.00)	0 (0.00)
Pruritus	2 (0.18)	0 (0.00)	1 (0.10)	0 (0.00)
Exanthema	0 (0.00)	0 (0.00)	0 (0.00)	0 (0.00)
Allergic reaction	0 (0.00)	0 (0.00)	0 (0.00)	0 (0.00)
Vomiting	0 (0.00)	0 (0.00)	0 (0.00)	0 (0.00)
Fever (>37.8ºC)	0 (0.00)	0 (0.00)	0 (0.00)	0 (0.00)

Supplementary Table S2. Frequency of non-immediate local and systemic adverse events by dose and schedule.

	First dose (n=2302)		Second dose (n=2212)		
	Schedule 0-14 (n=1090)	Schedule 0-28 (n=1212)	Schedule 0-14 (n=1038)	Schedule 0-28 (n=1174)	*Non-inferiority p value (a)*
**Local adverse events,** n (%)					
Pain	347 (31.8)	351 (29)	335 (32.3)	316 (26.9)	<0.001
Induration	22 (2.0)	36 (3.0)	52 (5.0)	27 (2.3)	<0.001
Pruritus	28 (2.6)	21 (1.7)	32 (3.1)	13 (1.1)	<0.001
Redness	27 (2.5)	14 (1.2)	24 (2.3)	11 (0.9)	<0.001
Swelling	17 (1.6)	19 (1.6)	31 (3.0)	26 (2.2)	<0.001
**Systemic adverse events,** n (%)					
Headache	282 (25.9)	311 (25.7)	224 (21.6)	236 (20.1)	<0.001
Fatigue	173 (15.9)	209 (17.2)	122 (11.8)	149 (12.7)	<0.001
Muscle pain	150 (13.8)	172 (14.2)	113 (10.9)	135 (11.5)	<0.001
Diarrhoea	106 (9.7)	95 (7.8)	64 (6.2)	73 (6.2)	<0.001
Nausea	62 (5.7)	69 (5.7)	39 (3.8)	48 (4.1)	<0.001
Anorexia	51 (4.7)	47 (3.9)	19 (1.8)	24 (2.0)	<0.001
Arthralgia	38 (3.5)	51 (4.2)	22 (2.1)	44 (3.7)	<0.001
Pruritus	23 (2.1)	12 (1.0)	18 (1.7)	8 (0.7)	<0.001
Exanthema	19 (1.7)	11 (0.9)	8 (0.8)	8 (0.7)	<0.001
Allergic reaction	15 (1.4)	7 (0.6)	8 (0.8)	9 (0.8)	<0.001
Vomiting	5 (0.5)	5 (0.4)	7 (0.7)	10 (0.9)	<0.001
Fever (>37.8ºC)	2 (0.2)	4 (0.3)	3 (0.3)	4 (0.3)	<0.001

(a) Non-inferiority test, with a margin of 15%, for comparing schedule 0-14 against 0-28.

Supplementary Table S3. Number of simultaneous not-immediate adverse events post vaccine administration by dose and schedule.

	First dose (n=2302)		Second dose (n=2212)	
	Schedule 0-14 (n=1090)	Schedule 0-28 (n=1212)	Schedule 0-14 (n=1038)	Schedule 0-28 (n=1174)
**Local adverse events, n (%)**				
0	746 (68.4)	857 (70.7)	700 (67.4)	857 (73.0)
1	304 (27.9)	305 (25.2)	276 (26.6)	276 (23.5)
2	23 (2.1)	37 (3.1)	34 (3.3)	27 (2.3)
3	12 (1.1)	10 (0.8)	17 (1.6)	10 (0.9)
≥4	5 (0.5)	3 (0.2)	11 (1.1)	4 (0.3)
**Systemic adverse events, n (%)**				
0	643 (59.0)	709 (58.5)	683 (65.8)	808 (68.8)
1	208 (19.1)	245 (20.2)	190 (18.3)	187 (15.9)
2	113 (10.4)	136 (11.2)	86 (8.3)	80 (6.8)
3	68 (6.2)	63 (5.2)	54 (5.2)	44 (3.7)
≥4	58 (5.3)	59 (4.9)	25 (2.4)	55 (4.7)

Supplementary Table S4. Duration of adverse events by dose and schedule.

	First dose				Second dose			
	Schedule 0-14		Schedule 0-28		Schedule 0-14		Schedule 0-28	
	n	Days, median (P10-P90)	n	Days, median (P10-P90)	n	Days, median (P10-P90)	n	Days, median (P10-P90)
**Local adverse events**								
Pain	347	2 (1 - 3)	351	1 (1 - 3)	335	2 (1 - 4)	316	2 (1 - 4)
**Systemic adverse events**								
Headache	282	2 (1 - 3)	311	1 (1 - 4)	224	1 (1 - 4)	236	2 (1 - 5)
Fatigue	173	2 (1 - 4)	209	1 (1 - 3)	122	2 (1 - 4)	149	2 (1 - 4)
Muscle pain	150	2 (1 - 4)	172	1 (1 - 4)	113	2 (1 - 5)	135	2 (1 - 5)

P10: 10th quantile; P90: 90th quantile

Supplementary Table S5. Frequency of adverse events by schedule, dose and age group.

	First dose (n=2302)		Second dose (n=2212)	
	Schedule 0-14 (n=1090)	Schedule 0-28 (n=1212)	Schedule 0-14 (n=1038)	Schedule 0-28 (n=1174)
**Local adverse events**				
Pain; n (%)				
<60 years	303 (37.9)	305 (37.4)	296 (39.3)	265 (33.5)
>=60 years	44 (15.2)	46 (11.6)	39 (13.7)	51 (13.3)
p value (a)	***<0.001***	***<0.001***	***<0.001***	***<0.001***
Induration; n (%)				
<60 years	18 (2.3)	25 (3.1)	45 (6.0)	22 (2.8)
>=60 years	4 (1.4)	11 (2.8)	7 (2.5)	5 (1.3)
p value (a)	*0.366*	*0.783*	*0.021*	*0.112*
Pruritus; n (%)				
<60 years	26 (3.3)	19 (2.3)	27 (3.6)	12 (1.5)
>=60 years	2 (0.7)	2 (0.5)	5 (1.8)	1 (0.3)
p value (a)	*0.018*	*0.023*	*0.130*	*0.072*
Redness; n (%)				
<60 years	23 (2.9)	12 (1.5)	21 (2.8)	9 (1.1)
>=60 years	4 (1.4)	2 (0.5)	3 (1.1)	2 (0.5)
p value (a)	*0.160*	*0.140*	*0.098*	*0.519*
Swelling; n (%)				
<60 years	14 (1.8)	12 (1.5)	24 (3.2)	17 (2.2)
>=60 years	3 (1.0)	7 (1.8)	7 (2.5)	9 (2.3)
p value (a)	*0.400*	*0.696*	*0.544*	*0.834*
**Systemic adverse reactions**				
Headache; n (%)				
<60 years	236 (29.5)	243 (29.8)	180 (23.9)	186 (23.5)
>=60 years	46 (15.9)	68 (17.2)	44 (15.5)	50 (13.0)
p value (a)	***<0.001***	***<0.001***	*0.003*	*<0.001*
Fatigue; n (%)				
<60 years	145 (18.1)	163 (20.0)	92 (12.2)	111 (14.1)
>=60 years	28 (9.7)	46 (11.6)	30 (10.6)	38 (9.9)
p value (a)	***0.001***	***<0.001***	*0.465*	*0.045*
Muscle pain; n (%)				
<60 years	125 (15.6)	138 (16.9)	78 (10.3)	103 (13.0)
>=60 years	25 (8.6)	34 (8.6)	35 (12.3)	32 (8.3)
p value (a)	*0.003*	***<0.001***	*0.361*	*0.018*
Diarrhoea; n (%)				

<60 years	87 (10.9)	77 (9.4)	56 (7.4)	61 (7.7)
>=60 years	19 (6.6)	18 (4.5)	8 (2.8)	12 (3.1)
p value (a)	*0.033*	*0.003*	***0.006***	*0.002*
Nausea; n (%)				
<60 years	54 (6.8)	55 (6.7)	33 (4.4)	34 (4.3)
>=60 years	8 (2.8)	14 (3.5)	6 (2.1)	14 (3.6)
p value (a)	*0.012*	*0.024*	*0.087*	*0.593*
Anorexia; n (%)				
<60 years	47 (5.9)	43 (5.3)	16 (2.1)	17 (2.2)
>=60 years	4 (1.4)	4 (1.0)	3 (1.1)	7 (1.8)
p value (a)	***0.002***	*<0.001*	*0.254*	*0.709*
Athralgia; n (%)				
<60 years	30 (3.8)	35 (4.3)	14 (1.9)	28 (3.5)
>=60 years	8 (2.8)	16 (4.0)	8 (2.8)	16 (4.2)
p value (a)	*0.43*	*0.840*	*0.338*	*0.598*
Pruritus; n (%)				
<60 years	18 (2.3)	11 (1.3)	15 (2.0)	5 (0.6)
>=60 years	5 (1.7)	1 (0.3)	3 (1.1)	3 (0.8)
p value (a)	*0.593*	*0.117*	*0.427*	*0.721*
Exanthema; n (%)				
<60 years	16 (2.0)	7 (0.9)	6 (0.8)	3 (0.4)
>=60 years	3 (1.0)	4 (1.0)	2 (0.7)	5 (1.3)
p value (a)	*0.282*	*0.756*	*0.999*	*0.123*
Allergic reaction; n (%)				
<60 years	13 (1.6)	5 (0.6)	6 (0.8)	6 (0.8)
>=60 years	2 (0.7)	2 (0.5)	2 (0.7)	3 (0.8)
p value (a)	*0.378*	*0.999*	*0.999*	*0.999*
Vomiting; n (%)				
<60 years	4 (0.5)	4 (0.5)	6 (0.8)	6 (0.8)
>=60 years	1 (0.3)	1 (0.3)	1 (0.4)	4 (1.0)
p value (a)	*0.738*	*0.999*	*0.681*	*0.737*
Fever (>37.8ºC); n (%)				
<60 years	2 (0.3)	3 (0.4)	3 (0.4)	4 (0.5)
>=60 years	0 (0.0)	1 (0.3)	0 (0.0)	0 (0.0)
p value (a)	*0.999*	*0.999*	*0.566*	*0.310*

Sample sizes for first dose. Schedule 0-14 <60 years n=800; schedule 0-14 >=60 years n=290; schedule 0-28 <60 years n=816; schedule 0-28 >=60 years n=396. Sample sizes for second dose. Schedule 0-14 <60 years n=754; schedule 0-14 >=60 years n=284; schedule 0-28 <60 years n=790; schedule 0-28 >=60 years n=384.

(a) For each scheme subgroup, AE differences by age group were compared using two-sided tests.

Supplementary Table S6. Incidence of COVID-19 positive patients by immunization schedule in different subpopulations.

	Schedule 0-14		Schedule 0-28		
	N Total	% COVID19	N Total	% COVID19	Non-inferiority p value*
Age (years)					
18-59	760	3.9	793	2.4	<0.001
60-98	285	1.8	387	1.3	<0.001
Sex					
Female	751	3.2	588	2.2	<0.001
Male	465	3.4	583	1.9	<0.001
Health setting workers					
Yes	421	2.4	290	1.7	<0.001
No	615	4.5	881	3.1	<0.001
Comorbidities					
>=1	465	3.0	531	2.3	<0.001
None	571	3.5	639	1.9	<0.001
Obesity					
Yes	194	2.6	261	3.4	<0.001
No	841	3.4	909	1.7	<0.001

Incidence of COVID-19 14 days post second dose or after.

Data presented as sample size for each schedule segmented by levels of sociodemographic characteristic and percentage of COVID-19 cases in each subgroup. *Non-inferiority test, with a margin of 15%, for comparing schedule 0-14 against 0-28.

(a) Two asymptomatic cases, three COVID-19 cases before 14 days post 2nd dose, and those inoculated out of vaccination window were omitted from this analysis.
