# Supplementary material for: Safety and Non-Inferiority Evaluation of Two Immunization Schedules with an Inactivated SARS-CoV-2 Vaccine in Adults: A Randomized Clinical Trial
Source: Vaccines (Basel). 2022 Jul 6;10(7):1082. doi: 10.3390/vaccines10071082 (PMC9323976; doi:10.3390/vaccines10071082)
Supplement: Supplementary file 1 [file vaccines-10-01082-s001.zip › vaccines-1768725-Supplementary_Appendix.pdf]

Supplementary Appendix

1. CoronaVac03CL Study Team

<u>Center CL1</u>: Áreas Ambulatorias Marcoleta - Pontificia Universidad Católica de Chile.

Álvaro Rojas, María Soledad Navarrete, Constanza Del Río, Dinely Del Pino, Natalia Aguirre, Grecia Salinas, Franco Vega, Acsa Salgado, Thomas Quinteros, Marlene Ortiz, Marcela Puente, Alma Muñoz, Patricio Astudillo, Nicole Le Corre.

<u>Center CL2</u>: Clínica San Carlos de Apoquindo - Red de Salud UC-Christus

Marcela Potin, Juan Catalán, Melan Peralta, Consuelo Zamanillo, Nicole Keller, Rocío Fernández, Sofía Aljaro, Sofía López, José Tomás González, Tania Weil, Luz Opazo, Paula Muñoz, Inés Estay, Miguel Cantillana, Liliana Carrera, Matías Masalleras.

<u>Center CL4</u>: Clínica Los Andes - Universidad de Los Andes

Paula Guzmán, Francisca Aguirre, Aarón Cortés, Luis Federico Bátiz, Javiera Pérez, Karen Apablaza, Lorena Yates, María de los Ángeles Valdés, Bernardita Hurtado, Veronique Venteneul, Constanza Astorga.

<u>Center CL5</u>: Clínica Alemana - Universidad del Desarrollo

Paula Muñoz-Venturelli, Pablo A. Vial, Andrea Schilling, Daniela Pavez, Inia Pérez, Amy Riviotta, Francisca González, Francisca Urrutia, Alejandra Del Río, Claudia Asenjo, Bárbara Vargas, Francisca Castro, Alejandra Acuña, Javiera Guzmán, Camila Astudillo.

<u>Center CL6</u>: Hospital Clínico Félix Bulnes - Universidad San Sebastián

Carlos M. Pérez, Pilar Espinoza, Andrea Martínez, Marcela Arancibia, Harold Romero, Cecilia Bustamante, María Loreto Pérez, Natalia Uribe, Viviana Silva, Bernardita Morice, Marco Pérez.

<u>Center CL7</u>: Hospital Dr. Gustavo Fricke - Universidad de Valparaíso

Marcela González, Werner Jensen, Claudia Pasten, M. Fernanda Aguilera, Nataly Martínez, Camila Molina, Sebastián Arrieta, Begoña López, Claudia Ortiz, Macarena Escobar, Camila Bustamante, Marcia Espinoza, Angela Pardo, Alison Carrasco, Miguel Montes, Macarena Saldías, Natalia Gutiérrez, Juliette Sánchez.

<u>Center CL8</u>: Hospital Carlos Van Buren- Universidad de Valparaíso

Daniela Fuentes, Yolanda Calvo, Mariela Cepeda, Rosario Lemus, Muriel Suárez, Mercedes Armijo, Shirley Monsalves, Constance Marucich, Cecilia Cornejo, Ángela Acosta, Xaviera Prado, Francisca Yáñez, Marisol Barroeta, Claudia López.

<u>Center CL9</u>: Complejo Asistencial Dr. Sótero del Rio

Paulina Donato, Martin Lasso, María Iturrieta, Juan Giraldo, Francisco Gutiérrez, María Acuña, Ada Cascone, Raymundo Rojas, Camila Sepúlveda, Mario Contreras, Yessica Campisto, Pablo González, Zoila Quizhpi, Mariella López, Vania Pizzeghello, Stephannie Silva.

2. Members of the Independent Data Safety Monitoring Committee.

Luis Delpiano, MD, Pediatric Infectious Diseases Specialist, Hospital San Borja Arriarán, Santiago, Chile.

Macarena Lagos, MD, Immunologist, Clínica Las Condes and Hospital Padre Hurtado, Santiago, Chile.

Gloria Icaza, MD, Epidemiologist and Statistician, Universidad de Talca, Talca, Chile.

Leonardo Chanqueo, MD, Infectious Disaeses Specialist, Hospital San Juan de Dios, Santiago, Chile.

Mónica Imarai, PhD, Universidad de Santiago, Santiago, Chile.
